# Viral sepsis – pathophysiology and disease manifestation

**DOI:** 10.1007/s15010-025-02486-z

**Published:** 2025-02-17

**Authors:** Lutz G Gürtler, Wolfgang Schramm, Rainer Seitz

**Affiliations:** 1 Max von Pettenkofer Institute - Virology, Ludwig-Maximilians University (LMU), München, Germany; 2 Rudolf Marx Foundation at the LMU, München, Germany; 3 Paul-Ehrlich-Institute, Federal Institute for Vaccines and Biomedicine, Langen, Germany

**Keywords:** Sepsis, Cytokine storm, Dengue virus, Ebola virus, SARS-CoV-2, Rabies virus

## Abstract

Viral infection is found in approximately 30% of all sepsis cases and may be followed by bacterial infection in organs such as the lungs. Sepsis manifests as fever, hemorrhagic lesions and cell death. Organ dysfunction caused by sepsis, such as meningitis and encephalitis, can lead to organ damage. Sepsis is induced by various viral components, host cells and cellular mediators, such as cytokines and chemokines. Cytokines are secreted from stimulated macrophages, monocytes, dendritic cells and T lymphocytes.

Further contributors to sepsis are the cleavage products after activation of the complement cascade with anaphylatoxin generation and peptides of the activated clotting cascade, thrombocytopenia and thrombocyte function alteration, intravasal clotting and/or endothelial leakage. The cells involved in viral sepsis are neutrophil granulocytes, monocytes and macrophages, dendritic cells and thrombocytes, and finally, endothelial cells and epithelial cells.

Prolonged cytokine release leads to cell damage, immune cell dysfunction and exhaustion, and either impairs or hyperactivates immune cells. The course of viral sepsis may be enhanced by some patient conditions including age, underlying diseases such as diabetes, obesity; and immunodeficiency. Viral sepsis, similar to bacterial sepsis, is an extremely complex disorder, and the involvement of the abovementioned cellular and humoral components can present quite divergent biological and clinical patterns.

Examples of viral sepsis discussed in the manuscript include three viruses causing Dengue fever – an emerging infection, COVID-19 – a disease with a prolonged course, Ebola disease – a disease with typically complete viral clearance, while rabies virus – induces a disease that causes coma and death before signs of viral sepsis are apparent.

## Characterization of sepsis

Sepsis may be caused by bacteria, fungi and viruses and is a life-threatening condition characterized by dysfunction of cells and dysregulation of immune cells, cell mediators and organs. Occurrence and severity are dependent on the infectious agent(s) and age, immune status and underlying disease of a patient. Globally, approximately 50 million patients develop sepsis annually [1–3].

### Viral sepsis

Viral sepsis is an organ dysfunction caused by viral infection and the dysregulation of the host response by virus components [4]. Crucial elements of viral sepsis are the initiated cytokine storm leading to cell activation, cell damage and cell death, advancing to organ damage with a high risk of mortality and a dysregulated immune reaction, leading to thromboinflammation [5, 6]. Thromboinflammation involves both microthrombosis and cell injury [6, 7]. Dysregulation in viral sepsis typically occurs in the clotting cascade and the associated activated thrombocytes and in complement activation and associated dendritic cells, monocytes, macrophages and neutrophils [8]. Viral sepsis is regional and seasonal, with an incidence of approximately 270 per 100,000 in the adult population [4]. Approximately 24–30% of all sepsis cases were virus induced during the period 2019 to 2020 of SARS-CoV-2 spread [9], with a high range of variability; however, in a 2019 study of community-acquired pneumonia patients, only 3% of sepsis cases were virus induced [10]. The outcome of viral sepsis is highly dependent on the initiating virus, the viral load and the patient’s health status [4]. As shown by investigations of patients in China infected with severe thrombocytopenia fever syndrome virus (STFSV), a Bandavirus and member of the Bunyaviridae family, the infection causes IFN-type I-related hyperinflammation caused by plasmablasts (plasmacytoid cells) and monocyte activation [11]. Viral infection may be followed by autoimmunity, as described by manifestation of type 1 diabetes and rheumatoid arthritis. The basis of autoimmune reactivity is molecular mimicry, epitope spreading to antigen-presenting cells, structurally altered immunogenic antigens from damaged cells and immortalization of some infected B cells [12].

### Bacterial sepsis

Bacterial sepsis accounts for approximately 60% of all sepsis cases, and an additional 5% are caused by fungal sepsis [1]. As sepsis may initially be virus induced and subsequently superinfected by bacteria, mixed infections are common. Bacterial sepsis is caused by gram-positive (60%) and gram-negative (40%) bacteria [1, 13]. Bacteria have long coevolved with the mammalian immune system in that lipopolysaccharide (LPS) and lipid A of gram-negative bacteria potently stimulate the complement cascade and immune defense by peptidoglycan, which is common in all bacteria [14]. Bacterial components such as lipid A are recognized by the myeloid differentiation factor MD-2, flagellin by TLR-5 (toll like receptor 5), and bacterial components by TLR-2, -4 and − 9. These receptors are expressed on myeloid cells, such as macrophages and neutrophils. After bacterial components bind to these receptors, T lymphocytes, B lymphocytes and NK cells (natural killer cells) are stimulated [15, 16]. Extensive stimulation leads to immune exhaustion, which is characterized by a progressive loss of function and reduced proliferation activity by chronic antigen stimulation of immune cells [1, 9, 15, 16]. Bacterial components such as LPS and peptidoglycan activate the clotting cascade and thrombocytes, force cytokine release [17, 18] and induce immunosuppression [19].

Sepsis-1 is characterized by a systemic inflammatory response against an infectious agent [20], and since 2016, sepsis-3 has been defined as severe infection with organ dysfunction and a dysregulated host response [20–22]. Clinical trials to mitigate the burden and reduce the mortality of bacterial sepsis by plasma exchange, cortisol or acetylsalicylic acid application have been fairly successful [7].

Bacterial sepsis is categorized by the SOFA score (sequential organ failure assessment), which rates cardiovascular, coagulation, liver, neurologic, renal and respiratory functions and is supported by several blood parameters, such as bilirubin and thrombocyte count. The SOFA score is further used to predict mortality [23, 24].

### Fungal sepsis

Fungal sepsis occurs mainly in neonates and immunosuppressed individuals and in patients with obstructive lung disease. The most frequently involved fungi are *Candida* sp. and *Aspergillus* sp., but all fungi may cause sepsis under specific conditions [25]. For diagnosis, a fungal component detectable in blood is 1,3-β-D-glucan, a carbohydrate of the cell wall that activates the innate immune system through TLR and C-type lectin [26]. The incidence is country and disease specific, affecting approximately 6.5 million annually, with a mortality of 3.8 million [27].

### Differences between bacterial and viral sepsis

Distinguishing bacterial and viral sepsis may be achieved using the following parameters: in bacterial sepsis, the causative bacteria are detected by culture, serologic tests or nucleic acid determination/amplification in blood or local lesions. Additionally, blood procalcitonin and CRP (C-reactive protein) levels are elevated, partially dependent on the time course of infection; further parameters are a high blood neutrophil granulocyte count and a low thrombocyte count [28, 29]. In patients with viral sepsis, blood parameters related to bacterial growth are negative. Distinguishing both types of sepsis is facilitated by the quantification of plasma proteins, such as antithrombin III for viral infection and ceruloplasmin for bacterial infection, and the quantification of neutrophils [30, 31]. The parameters of the activation of the complement and clotting cascades are present in both types of sepsis.

## Cytokine action

### Characterization of cytokines and chemokines

Cytokines are small proteins with molecular weights between 8 and 68 kDa that regulate communication between cells and their differentiation and proliferation, whereas chemokines are proteins with a higher molecular weight, 75–125 kDa, that build various loops by cysteine bridges. One of the main actions of cytokines is to influence the regulatory balance of cells of the innate and humoral immune systems and stimulate these cells during inflammation locally and in the whole organism [32]. Cytokines may be classified into different families, as γc is composed of IL-2, IL-4, Il-7, IL-9, IL-15, IL-21 and interferons (IFNs). By binding to receptors, cytokines contribute to lymphocyte development, maturation and differentiation and contribute to allergy, autoimmunity, immunodeficiency and cancer development [33, 34]. After cytokines bind to their receptors, JAK (Janus kinase) 1 to 3 and STAT (signal transducer and activator of transcription) 1 to 6 initiate gene expression, which is followed by cell activation, including immune cell activation to malignant hyperactivation [34].

### Action of interferons during viral sepsis

**Fig. 1:**
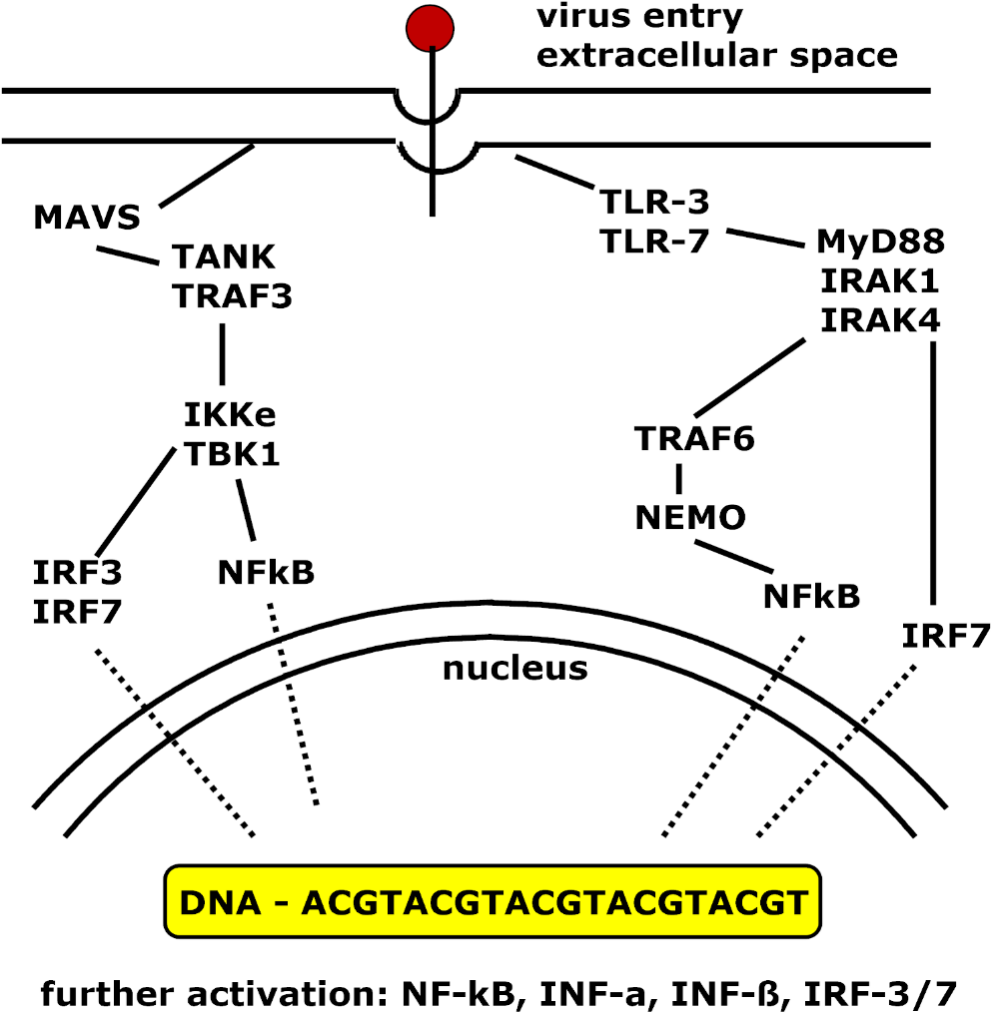
Scheme of the activation and modulation processes following virus attachment to a cell. After the virus binds, some mediators are replaced in the nucleus, leading to the synthesis of NF-κB, INF-α and IFN-β, IRF-3 and 7 and other mediators. Figure adapted from Franco et al. [35]. Abbreviations: INF – interferon; IRAK – interleukin-1 receptor-associated kinase; IRF-interferon regulatory factor; IKKe - I kappa B kinase epsilon; MAVS – mitochondrial antiviral signaling; MyD88 – myeloid differentiation primary response 88; NEMO – NF-κB essential modulator; NF-κB – nuclear factor kappa B; TANK – TRAF-associated NF-κB activator, a negative regulator of TLR; TBK1 – TANK binding kinase 1, a restrictor of the inflammatory response; TLR – Toll-like receptor; TRAF – tumor necrosis factor receptor-associated factor

In addition to cytokines toxins and metabolites liberated by a virus, fragments of lysed cells and their degraded components contribute to viral pathogenicity, leading to lesions of epithelial cells, endothelial cells, immune cells and other cells, which are complicated by organ failure and partially enhanced by hyperactivation of the immune system and death [32]. Interferons (IFNs) are essential cytokines because of their cell activation via STAT1 action [34, 35]: IFN type I is composed of IFN-α, which has multiple isoforms and is secreted by plasmacytoid and dendritic cells. IFN-β, which is secreted by all cell types, is a single protein. IFN type II consists of one cytokine, IFN-γ, which is secreted by natural killer cells. IFN type III has multiple isoforms, named IFN-λ, which are secreted by epithelial cells. A rough scheme of cytokine and interferon release after virus entry at the cellular level is shown in Fig. 1.

### IL-6 and IL-11 as examples of the diversity of cytokine action

Both IL-6 and IL-11 are related and important mediators of viral sepsis; they are members of the same family but exhibit different types of cell and tissue activation. Each interleukin uses a unique receptor that is present on different cell types. When IL-6 binds to immune cells, JAK-STAT3 signaling is activated [36]. When IL-11 attaches to its receptor on fibroblasts and stroma cells, ERK (extracellular regulated kinase) signaling ensues [37]. In addition to immune response dysregulation, IL-6 signaling influences further mental attention and initiates autoimmunity [38]. IL-11 is also involved in the pathology of aging [39]. The involvement of both interleukins in activation of non-immune cells shows that interleukin action goes far beyond a viral response. IL-11 is further involved in fibrosis and thrombocyte generation [40].

### Cytokine storm

There is no simple definition of a cytokine storm, but it describes a life-threatening situation of a systemic inflammatory syndrome caused by circulating cytokines that is aggravated by hyperactivation of immune cells [32]. Cytokine storms are known to result from severe influenza virus infection and bacterial sepsis, such as that caused by *Yersinia pestis*-induced plague [32]. The clinical signs of cytokine storm syndrome, also termed hyperinflammatory syndrome, are elevated temperature ≥ 38.5 °C, increased heartbeat and breathing frequency, hemorrhagic lesions, irritation of nerve cells leading to meningitis or encephalitis and arthritis. Clinical symptoms may even be more deleterious when endogenous CMV (cytomegalovirus) and EBV (Epstein-Barr virus) are reactivated [41].

### Cellular receptors essential for the defense reaction

Viruses and other pathogens are recognized by the cellular receptors PRRs (pattern recognition receptors), which are present in most cells and cell compartments [42, 43]. PRRs interact with PAMP (pathogen-associated molecular pattern) as viral nucleic acid, bacterial LPS, and fungal 1,3-β-D-glucan. The receptors are compiled in Table 1.

**Table 1:** Groups of cellular pattern recognition receptors (PRRs)

ALR	AIM2 like receptor; the protein AIM2 means: absent in melanoma 2
CLR	C-type lectin receptor
NLR	NOD like receptor; NOD means: nucleoside oligomerization domain
RLR	RIG-like receptor; RIG means: retinoic acid inducible gene
TLR	toll-like receptor

### Binding of viral components to PRR and TLR

The binding of viral components to PRRs leads to conformational changes in the structure of proteins and the activation of the NF-κB (nuclear factor kappa B) signaling cascade [42]. The binding of viral nucleic acid leads to receptor dimerization and the involvement of adaptor proteins such as MyD88 (myeloid differentiation primary response 88; a myeloid cell differentiation marker). In the cytosol, a complex of MyD88 and IRAK (interleukin receptor associated kinase) molecules assembles and activates NF-κB, followed by cytokine release, and IRF3/7 (interferon regulated factor) stimulates IFN type I production [42], as shown in Fig. 1.

Four TLRs are involved in viral nucleic acid binding: viral ds RNA binds to TLR-3, ss-RNA binds to TLR-7 and − 8, and all types of viral DNA bind to TLR-9. The attachment of viral nucleic acid to TLRs leads to the phosphorylation of NF-κB, which is subsequently translocated to the cell nucleus, followed by the release of cytokines such as TNF-α (tumor necrosis factor alpha), IL-2, IL-6, IL-8 and IL-12, leading to cell activation [35].

### Balancing the upregulation and suppression of cell activation

The upregulation of cytokine release is inhibited by TRAF family-associated NF-κB activator, whereas TRAF is a tumor necrosis-associated factor, which is a negative regulator of Toll-like receptor (TLR) signaling and suppressor of cell activation [35, 42], as shown in Fig. 1. A further mechanism to hinder self-cell destruction by TLR activation is the nonlinearity of signal transduction, which is triggered only after a certain threshold is reached, independent of the level of PAMP cellular response induction [42].

### TLR-related myddosome assembly

**Fig. 2:**
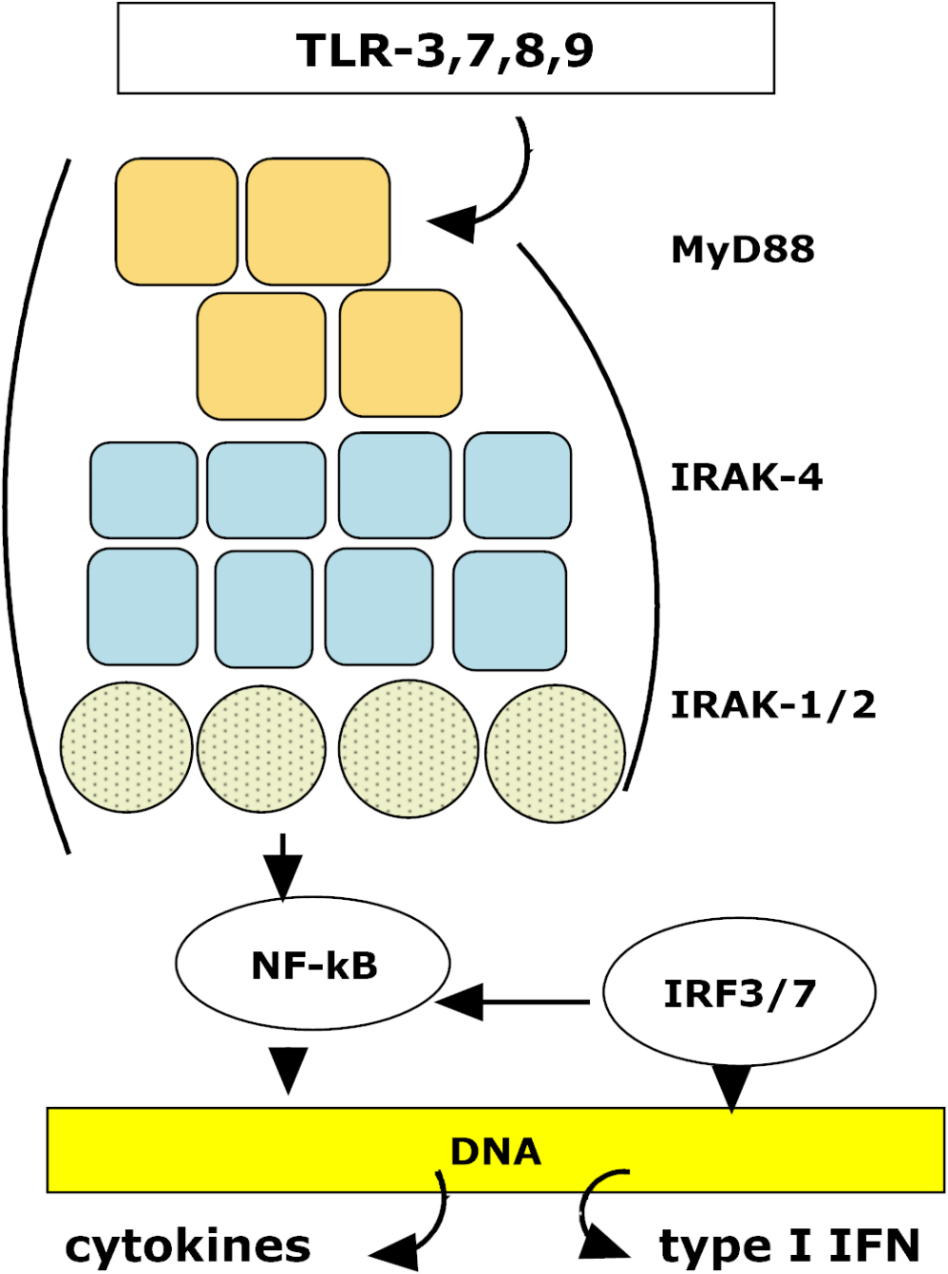
The Myddosome is formed by protein aggregation of at least 2 layers of MyD88, with two layers of IRAK-4 and one layer of IRAK-1 or -2. The aggregate complex is transported to the nucleus, and translocated NF-κB attaches to cellular DNA and induces cytokine expression. This process is supported by IRF3 and IRF7, which induce type I IFN expression. Data are according to the schemes of Bonhomme et Poirier [42] and Balka et De Nardo [46]. Abbreviations: IRAK-1 - interleukin-1 receptor-associated kinase; IRF - interferon regulatory factor; MyD88 - myeloid differentiation primary response 88; NF-κB - nuclear factor kappa B; TLR - Toll-like receptor

Myddosomes are described as oligomeric complexes of MyD88 and IRAK1/2 and IRAK4 molecules that are formed intracellularly after viral component and bacterial LPS induced TLR activation and IL-1R stimulation [44]. TLR activation by viral nucleic acid leads to cytokine release [42, 45] and aggregation of intracellular oligomeric signaling complexes [46, 47]. MyD88 is not involved in signal transduction as it forms only short oligomers to prevent nonpathogenic stimulation [42]. A scheme of Myddosome formation associated with cytokine and type I IFN synthesis is shown in Fig. 2.

## Cells involved in viral sepsis

**Plasmacytoid dendritic cells** control apoptosis, generation, proliferation and maturation of megakaryocytes by secretion of type I IFN, type III IFN, TNF-α and the mediators of inflammation MyD88 and IRF7, which are the main contributors to viral sepsis [15, 48]. The activation of plasmacytoid cells is driven by TLR7/9, with a possible risk of autophagy and autoimmunity and the induction of chronic disease, as described during HCV and EBV infection [49].

**Monocytes**,** dendritic cells and macrophages** during viral sepsis react with early signaling after the binding of viral nucleic acid to TLRs [46]. These cells phagocytose the virus, mostly by clathrin-mediated endocytosis, and cleave it into components that are expressed on the cell surface. Antigen presentation by dendritic cells and macrophages may be inhibited by regulatory T cells, TGF-β (transforming growth factor beta) and IRF4, which leads to impairment of the immune response [12, 16].

### Thrombocytes

in addition to their contribution to clot formation, thrombocytes contribute to antiviral activity through the secretion of kinocidin peptides; the expression of TLR, complement and immunoglobulin receptors; and the release of cytokines and chemokines, which is referred to as thromboinflammation [5, 6, 51].

**Fig. 3:**
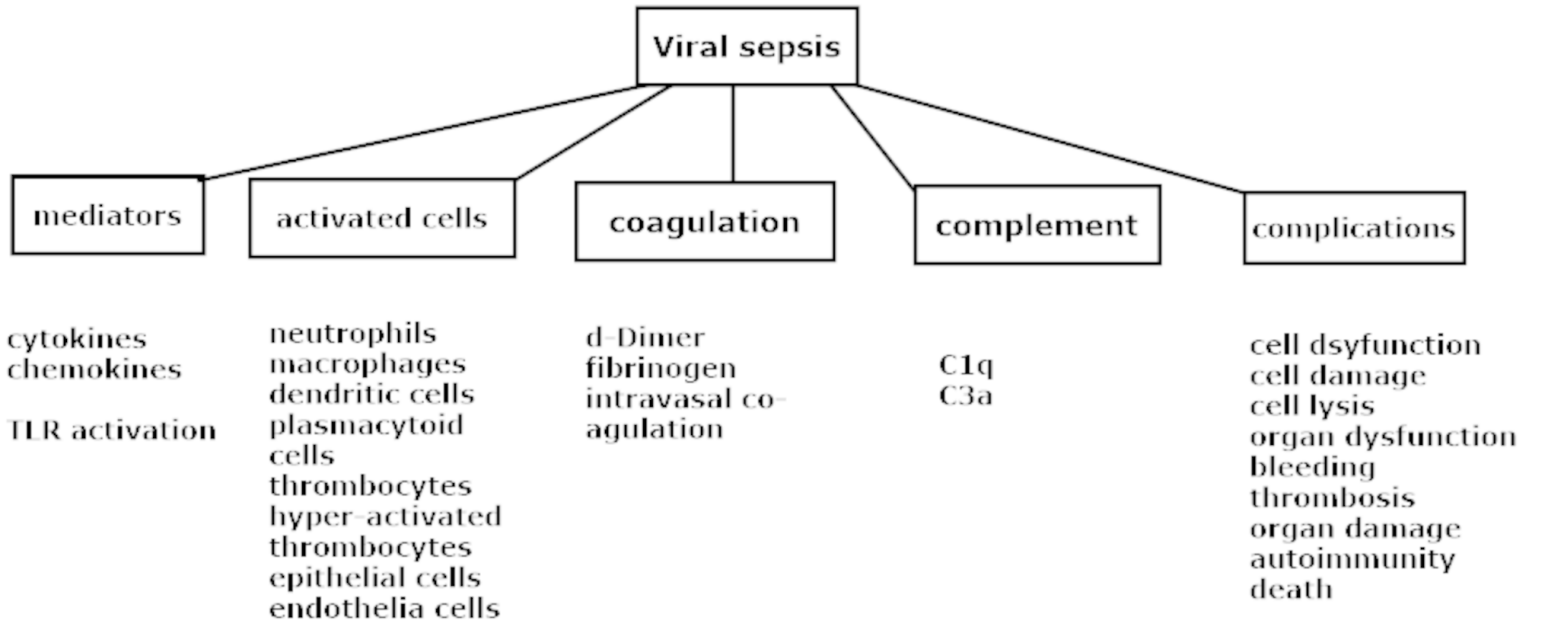
Overview of the main events associated with viral interference leading to sepsis

**Neutrophils** are involved after lysis through the liberation of granule components such as elastase, myeloperoxidase and cathepsin G; the formation of NETs (neutrophil extracellular traps); and the establishment of nucleic acid complexes that are enlarged by the attachment of cellular chromatin [28, 50, 51]. For example, influenza virus is a known trigger of NETosis (NET formation), which involves activation of stimulator of interferon genes (STING) and the complement cascade and increasing tissue factor levels [52]. NETs support virus clearance by their binding to nucleic acids, cleaving viral surface components, and increasing the phagocytosis of viruses by macrophages and dendritic cells and primed lymphocyte generation [53, 54], as shown in Fig. 3.

Another contributor to viral clearance is the formation of **extracellular vesicles** (EVs): these tissue factor (TF)-containing particles are released from activated cells, such as monocytes, and are found during viral sepsis caused by HIV, Ebola virus and SARS-CoV-2 [55]. The formation of high amounts of EVs is associated with increased mortality [55].

The figure shows the mediators, activated cells and cleavage products of the complement and clotting cascade activation. Alterations at the cellular and organ levels by activation, dysfunction and organ damage result in increased mortality.

## Examples of viruses that lead to viral sepsis

Three viruses that induce sepsis are described, and the pathophysiology and clinical symptoms of each individual virus differ. Furthermore, the pathogenic action of the rabies virus, which is lethal for humans without causing signs of viral sepsis before disease manifestation, is discussed. The modes of transmission and defense reaction are shown in Table 2.

**Table 2:** Main characteristics of the four viruses discussed

Virus	Transmission	Immunity	Prevention, besides hygiene
Dengue	Mosquito bite	Complete, but ADE^*^	Vaccines for children
SARS-CoV-2	Aerogene, direct contact	Partially incomplete Short duration of immunity	Frequent variant adapted vaccination
Ebola	Direct contact, bat, human, monkey	Complete, very rarely incomplete	Vaccination, possibly treatment
Rabies	Bite by rabietic dog or fox	Only after vaccination	Vaccination, bite prevention

^*^Abbreviation: ADE – antibody-dependent enhancement

### Dengue virus (DENV)

DENV is a member of the flavivirus group that is spread by mosquito bites of *Aedes albopictus* and *Aedes aegypti* in tropical and subtropical zones worldwide. The infectious burden is 50–400 million infections annually [56], and most of the infected individuals remain asymptomatic. Approximately 96 million develop disease [57] with signs of hemorrhagic fever, which may be complicated by DSS, i.e., dengue hemorrhagic syndrome [58]. DENV is divided into 4 antigenically different serotypes, with DENV-2 and − 4, and DENV-1 and DENV-3 being more closely related. Complete immunity is usually established after repeated infection, leading to cross-reactivity with other serotypes [59].

Virulence is based on an increase in the levels of the cytokines IL-2, -4, -6, -8 and − 10, TNFα and IFN-γ and a Th2 lymphocyte response. IL-8, IL-10 and TNFα regulate monocyte and macrophage activation and endothelial cell damage, and associated coagulation disorders lead to bleeding [60, 61].

A further nonviral but immune system-related pathogenic complication is antibody-dependent enhancement (ADE), which leads to rapid attachment and entry of the DENV-antibody-coated virus to Fc-receptor-equipped cells [60] when cross reactive but not neutralizing antibodies bind to DENV. ADE is found in young children who still have a certain level of maternal antibodies or after infection with another DENV serotype.

Therapy: A drug that reduces replication has been recently developed [57, 58]. Two vaccines are available, one by Sanofi (Dengvaxia^®^) and a second by Takeda (Qdenga^®^), which are life-attenuated virus combinations. The application is restricted to children, and fear of the induction of ADE by vaccination is not completely excluded.

### Severe acute respiratory syndrome coronavirus 2 (SARS-CoV-2)

Similar to DENV and Ebola virus, SARS-CoV-2 is a zoonotic disease that changed its epidemiologic distribution after 2019 when the human pandemic started and the disease COVID-19 was characterized [62]. The majority of infected individuals present with no or minor unspecific signs of disease; however, in 10% of those infected, risk factors such as hypertension, obesity, diabetes, advanced age and immunosuppression may contribute to life-threatening acute pulmonary distress syndrome (ARDS) and kidney and heart failure. COVID-19 may result in a chronic infection defined as long COVID [9, 63, 64]. SARS-CoV-2 sepsis includes thromboinflammation, with a risk of severe frailty and death [64–66].

Fibrinogen thrombocyte aggregates are induced by the spike protein S1 and complement component C3a, leading to vascular C3a deposits as a first step of thrombus formation [67]. The immune reaction might be enhanced by high expression of HLA proteins [68]. Viral load levels in nasopharyngeal secretions and blood may be correlated with the severity of symptoms [49, 69]. Long COVID develops in approximately 10% of COVID-19 cases [70, 71]. Symptoms of viral sepsis may be enhanced by reactivated viruses [70]. Presently, more than 1,100 publications of viral sepsis and COVID-19 are cited in PubMed.

Therapy: When given at the beginning of SARS-CoV-2 replication, drugs such as Paxlovid^®^ (nirmatrelvir/ritonavir) or Lagevrio^®^ (molnupiravir, not reported in Germany) reduce the viral load and mitigate symptoms [63]. Vaccination: there are numerous vaccines available that are partially strain specific adapted, partially confected by self-replicating mRNA in liposomes [64]. Only 5 vaccines are presently notified in Germany, with a total of 19 in Europe.

### Ebola virus

Ebola and Marburg virus are filoviruses that can induce viral sepsis and are mainly found in bats and pteropoids in tropical zones [72–74]. These viruses can occasionally be transmitted to other animals and humans and filoviruses are adapted to the mammalian immune system. The soluble glycoprotein of Ebola virus contributes the most to viral sepsis, and its delta peptide acts as an enterotoxin [72]. Some infected individuals remain clinically asymptomatic or paucisymptomatic. The severe clinical course is characterized by diarrhea with fever, weakness and fatigue; bleeding by endothelial cell layer leakage; and disseminated intravascular coagulation (DIC) [75]. The range of virus strain-dependent death rates is 30–90% [76, 77]. In a few survivors, the virus will persist in all infected organs, such as the nervous system, kidney and genital tract, and replicating viruses can be transmitted to sexual partners and by breast feeding [75, 76].

In 2010, it was reported that approximately 20% of the rural population in Gabon had antibody titers that cross-reacted with Zaire Ebola virus, but these individuals had never suffered from hemorrhagic fever [78]. Bombali virus is closely related to Ebola virus, Tai-forest virus and Reston virus, but it is apathogenic for humans, as is Reston virus in Southeast Asia [51]. Bombali virus was identified in 2018, is prevalent in Central Africa and induces Ebola virus cross-reactive antibodies but not viral sepsis [79]. Whether a previous infection by Bombali virus protects against Ebola virus infection is unknown but seems likely.

Therapy: Remdesivir inhibits viral replication, and 2 monoclonal antibodies are available. Vaccination: The first vaccines were configured as a recombinant glycoprotein on a vesicular stomatitis virus (VSV) backbone or on a modified virus Ankara (MVA) basis [80], and an additional vaccine that is configurated on self-replicating mRNA in liposomes [81].

## Rabies virus (RABV), a virus that causes death without viral sepsis

RABV is a deadly disease in unvaccinated humans. The virus may persist in initially infected epithelial or muscle cells up to 12 months after the bite. In these cells RABV coated by a membrane, is forming a vesicle, which is transported retrogradely in the axons of nerves to the brain. During transport, no immunogenic RABV proteins are expressed on the surface of nerve cells [82, 83]. Thus, viral sepsis is not initiated, and a specific immune reaction starts only after RABV replicates to high amounts (10^9^ per g brain tissue) in the brain by lysis of astrocytes, microglia and nerve cells [84]. In the brain, intracellularly produced virus is concentrated in liquid organelles, Negri bodies, which also contain heat shock protein 70 (Hsp70) [85]. Within 2‒3 weeks, RABV spreads from the brain throughout the whole organism, including to skin and saliva and reaches viral loads of 10^6^/mL in saliva [86].

Cytokine level alterations and cell damage: The first host cellular response after virus release is the activation of IFN-α and IFN-β, but RABV proteins antagonize IFN action by inhibiting IFN production and the migration of dendritic cells [82, 83]. After RABV has reached the brain, IFN-λ induces the secretion of IL-17 and CXCL10 (previously named IP-10 – IFN-γ-induced protein 10), which enhances BBB (blood–brain barrier) penetration by RABV in both directions [87]. Coma and death are ultimately caused by the destruction of brain cells [88].

Therapy: No drugs are available that can successfully treat rabies because the diagnosis of rabies is usually made when the paralytic or furious state is evident, at which time any treatment would be instituted too late and thus not be effective, with patients dying within 8 days [89]. Vaccines are based on inactivated purified cell culture-grown virus and can be applied for prophylaxis, pre-exposure and post-exposure vaccination [90].

## Conclusion

Viral sepsis is a frequent event during severe viral infection when the initial rapid immune response by the innate or acquired immune system is not sufficiently high to prevent viral replication and spread. The initial defense reaction is supported by high cytokine release and the intracellular activation of NFκB and interferon secretion, and further through the contributions of neutrophils, thrombocytes, plasmacytoid cells, dendritic cells, macrophages and endothelial cells. Additionally, the activation of the clotting and complement cascades supports virus elimination by cell lysis and increased phagocytosis, thus preventing organ damage but with a certain risk of inducing an autoimmune reaction. Autoimmunity remains a side effect of an intense immune reaction in predisposed individuals. Excessive viral toxicity and reduced immune defense are hazardous and may be complicated by subsequent bacterial infection. Therapeutic applications, such as IFN-α, IFN-β or IL-6 inhibitors, were frequently not potent enough to change the course of viral sepsis. Extended knowledge of the pathophysiology of viral sepsis is needed to modulate cytokine interference and support immune defense and may contribute to restoring health.

## Data Availability

No datasets were generated or analysed during the current study.
